# CLMT: graph contrastive learning model for microbe-drug associations prediction with transformer

**DOI:** 10.3389/fgene.2025.1535279

**Published:** 2025-03-12

**Authors:** Liqi Xiao, Junlong Wu, Liu Fan, Lei Wang, Xianyou Zhu

**Affiliations:** ^1^ College of Computer Science and Technology, Hengyang Normal University, Hengyang, China; ^2^ Technology Innovation Center of Changsha, Changsha University, Changsha, China; ^3^ Hunan Engineering Research Center of Cyberspace Security Technology and Applications, Hengyang Normal University, Hengyang, China

**Keywords:** microbe-drug association, graph transformer, similarity matrices, contrastive learning, nonlinear relationships, prediction accuracy, graph augmentation

## Abstract

Accurate prediction of microbe-drug associations is essential for drug development and disease diagnosis. However, existing methods often struggle to capture complex nonlinear relationships, effectively model long-range dependencies, and distinguish subtle similarities between microbes and drugs. To address these challenges, this paper introduces a new model for microbe-drug association prediction, CLMT. The proposed model differs from previous approaches in three key ways. Firstly, unlike conventional GCN-based models, CLMT leverages a Graph Transformer network with an attention mechanism to model high-order dependencies in the microbe-drug interaction graph, enhancing its ability to capture long-range associations. Then, we introduce graph contrastive learning, generating multiple augmented views through node perturbation and edge dropout. By optimizing a contrastive loss, CLMT distinguishes subtle structural variations, making the learned embeddings more robust and generalizable. By integrating multi-view contrastive learning and Transformer-based encoding, CLMT effectively mitigates data sparsity issues, significantly outperforming existing methods. Experimental results on three publicly available datasets demonstrate that CLMT achieves state-of-the-art performance, particularly in handling sparse data and nonlinear microbe-drug interactions, confirming its effectiveness for real-world biomedical applications. On the MDAD, aBiofilm, and Drug Virus datasets, CLMT outperforms the previously best model in terms of Accuracy by 4.3%, 3.5%, and 2.8%, respectively.

## 1 Introduction

The human body hosts trillions of microorganisms, including bacteria, archaea, fungi, protozoa, and viruses, collectively forming the human microbiota, which interacts closely with its host (Gevers et al., 2012; Sommer and Bäckhed, 2013). These microorganisms inhabit various regions such as the skin, oral and nasal cavities, gastrointestinal tract, and genitourinary system, exerting profound effects on health. For instance, they regulate gastrointestinal function, support internal balance, and facilitate metabolic activities (Gill et al., 2006; Ventura et al., 2009). Additionally, the microbiota collaborates with mucosal barriers to prevent pathogen invasion (Macpherson and Harris, 2004). Microbes also contribute to processes like sugar metabolism and vitamin synthesis, both critical for T-cell response (Kau et al., 2011). However, an imbalance in microbial populations, or dysbiosis, can lead to conditions such as diabetes (Wen et al., 2008), inflammatory bowel disease (Durack and Lynch, 2019), and even cancer (Schwabe and Jobin, 2013). Furthermore, pathogens like certain bacteria and viruses are linked to numerous infectious diseases, including pneumococcal pneumonia, with evidence suggesting involvement in up to 27 conditions (Wang D. et al., 2020). The overuse and misuse of medications in recent years have accelerated microbial resistance, creating significant obstacles for clinical treatments and drug development. Microbial metabolism also influences drug efficacy, absorption, and toxicity, highlighting its critical role in pharmacology (Zimmermann and Curtis, 2019; McCoubrey et al., 2023). For example, interactions between intestinal flora and anticancer drugs can alter therapeutic outcomes and side effects. Strategies such as probiotics, prebiotics, synbiotics, biologics, and antibiotics have been proposed to manage microbial populations and enhance treatment effectiveness (Panebianco et al., 2018). Consequently, identifying microbe-drug relationships is a vital challenge in precision medicine, underscoring the urgent need for advanced computational models to explore these interactions.

In recent years, the rise of microbial resistance has paralleled the increasing diversity of drug candidates explored by the medical community (Jiang et al., 2024). Traditional pharmaceutical research often relied on cultivating specific microbial populations under controlled conditions before integrating them into drugs, a process that is both time-intensive and laborious. This challenge underscores the pressing need for advanced computational methods to identify potential microbe-drug relationships, which could revolutionize drug discovery and disease diagnosis (Jiang et al., 2023; Jiang et al., 2025). The advent of bioinformatics has facilitated the establishment of several databases documenting experimentally validated microbe-drug associations, including MDAD (Sun et al., 2018), aBiofilm (Rajput et al., 2018), and DrugVirus (Andersen et al., 2020).

To complement these resources, numerous computational approaches have emerged. For instance, HMDAKATZ, developed by (Zhu et al., 2019), utilizes KATZ metrics within a heterogeneous network to predict microbial-drug correlations. However, its applicability is limited for novel drugs without known microbial associations or isolated microbes lacking disease links. Similarly (Long et al., 2020a), introduced EGATMDA, a graph attention network-based framework with hierarchical attention mechanisms for analyzing microbial-drug interactions. Despite its innovation, this model’s accuracy is constrained by its reliance on pre-existing association data for similarity computation.

Another approach, WHGMF, proposed by Ma and Liu (2022), employs weighted hypergraph learning with generalized matrix decomposition to estimate potential microbe-drug interactions. Yet, it overlooks critical biological details, such as microbial sequences and drug side effect-based similarities, which diminishes prediction accuracy. GCNMDA, introduced by Long et al. (2020a), combines graph convolutional networks and conditional random fields with an attention mechanism to predict microbial-drug associations. Nevertheless, its performance is hindered by noise within extracted similarity features.

Deng et al. (2022) presented Graph2MDA, which uses multimodal attribute graphs and a variogram self-encoder to analyze node-level information and infer potential interactions. In contrast (Tan et al., 2022), proposed GSAMDA, a model integrating graph attention networks with sparse self-encoders to compute microbe-drug correlations. However, GSAMDA struggles with sparse data matrices, limiting its effectiveness. Although these computational models exhibit strengths in certain areas, each faces distinct challenges, emphasizing the need for continued innovation in this field.

In binary relation prediction, selecting appropriate negative samples is critical for effective model training. However, identifying informative negative samples from a pool of candidate negatives remains a significant challenge (Li et al., 2022). This issue is particularly evident in link prediction tasks, where generating meaningful negative samples has long been a persistent problem. Conventional machine learning methods typically classify known associations between entities (labeled samples) as positive samples, while unrecognized or unlabeled associations are treated as candidate negatives (Yang et al., 2012). Yet, due to the scarcity of known microbe-drug associations in publicly available datasets, the imbalance between positive and negative samples becomes a critical issue. To mitigate this imbalance and preserve model performance, advanced negative sampling strategies are essential.

The most widely used approach, random sampling, involves selecting a subset of negative samples equal in number to the positive samples (Lou et al., 2022). While straightforward, this method often fails to prioritize informative negatives and may include irrelevant or noisy examples (López et al., 2013). Efforts to enhance negative sampling strategies (Zeng et al., 2020; Wei et al., 2021; Dai et al., 2022) have achieved limited success, as they do not sufficiently focus on identifying the most valuable negatives critical for effective classifier training. This oversight can result in undertraining and reduced predictive performance.

To address these limitations, we developed a novel microbe-drug association prediction model, CLMT. This model leverages a Graph Transformer network to identify potential associations between graph nodes. It incorporates contrastive learning and employs a four-phase approach with diverse augmented views as positive samples, significantly enhancing prediction accuracy. The key contributions of our work are as follows:

(1) We develop a novel heterogeneous graph-based model that employs a Graph Transformer network to effectively capture complex interactions between microbes and drugs. This allows the model to leverage long-range dependencies within the network structure, surpassing traditional GCN-based methods.
(2) We introduce contrastive learning into microbe-drug association prediction, a technique previously underexplored in this domain. The model generates multiple augmented graph views through node perturbation, treating them as positive samples, while negative samples are selected from different graphs. This contrastive loss mechanism significantly enhances the model’s ability to learn discriminative and generalizable embeddings.
(3) We conduct extensive experiments on three widely used public datasets (MDAD, aBiofilm and Drug Virus), demonstrating that CLMT significantly outperforms state-of-the-art prediction methods. We further validate CLMT’s ability to uncover novel microbe-drug associations through case studies on two common drugs, reinforcing the model’s practical value in biomedical research.

## 2 Materials and methods

### 2.1 Datasets

In this study, we used three publicly available datasets for model training and validation: the Microbe-Drug Association Database (MDAD), the aBiofilm database, and the Drug Virus database.

MDAD is a comprehensive resource specializing in known associations between microbes and drugs, integrating data from authoritative sources such as DrugBank, the Human Microbiome Project (HMP), KEGG, and PubChem. Specifically, the MDAD database includes 2,470 clinically or experimentally validated associations between 1,373 drugs and 173 microorganisms. Each association is backed by high-quality data and confirmed through rigorous experimental validation or clinical trials.

The aBiofilm database contains 2,884 associations between 1,720 drugs and 140 microorganisms, focusing on biofilm-associated microbial-drug interactions. It collects a substantial amount of experimental data, particularly on drug-microbe associations related to biofilm formation and inhibition.

The Drug Virus database provides an extensive collection of drug-virus interactions, which are critical for understanding the potential antiviral effects of drugs. This dataset integrates data from multiple biomedical resources, including DrugBank, CTD, and literature-reported associations, and contains over 3,000 drug-virus interactions covering a wide range of viral pathogens. The inclusion of this dataset allows us to assess the model’s ability to handle a broader spectrum of drug-target interactions, particularly in the context of antiviral drug discovery and drug repurposing.

To ensure the reliability of the analyzed results and the biological significance of the associations, we further incorporated drug-disease and microbe/virus-disease association data. The results of the analyses of the MDAD, aBiofilm, and Drug Virus datasets are presented in Table 1.

**TABLE 1:** Results of MDAD and aBiofilm dataset analysis.

Data set	Number of drugs	Microbial population	Number of diseases	Number of associations	Number of drug-disease associations	Number of microbe-disease associations
MDAD	1,373	173	109	2,470	1,121	402
aBiofilm	1,720	140	72	2,884	435	254
Drug Virus	1,950	200+	85	3,050+	720	580

Consistent with the methodology described by Tan et al. (2022), we implemented the following data screening strategy. First, we selected diseases that were associated with at least one drug and one microorganism in the MDAD dataset. This screening step yielded 109 diseases linked to both drugs and microorganisms. From these, we further extracted 1,121 drug-disease associations and 402 microbe-disease associations.

Similarly, we screened the aBiofilm dataset for diseases associated with at least one drug and one microorganism. This process identified 72 diseases, from which we extracted 435 drug-disease associations and 254 microbe-disease associations.

For the Drug Virus dataset, we applied the same screening criteria, selecting diseases associated with at least one drug and one virus. This step identified 85 diseases, from which we extracted 720 drug-disease associations and 580 virus-disease associations. The inclusion of the Drug Virus dataset allows us to evaluate the model’s performance on a larger and more diverse dataset, particularly in the context of antiviral drug discovery and cross-domain generalization.

By integrating the Drug Virus dataset into our study, we aim to assess the model’s scalability and robustness when applied to a broader range of biomedical problems. Additionally, given the growing need for antiviral drug repurposing—particularly in response to emerging viral diseases—this dataset provides an important benchmark for evaluating the model’s ability to predict drug-virus associations with potential clinical relevance.

### 2.2 Overview

**FIGURE 1:**
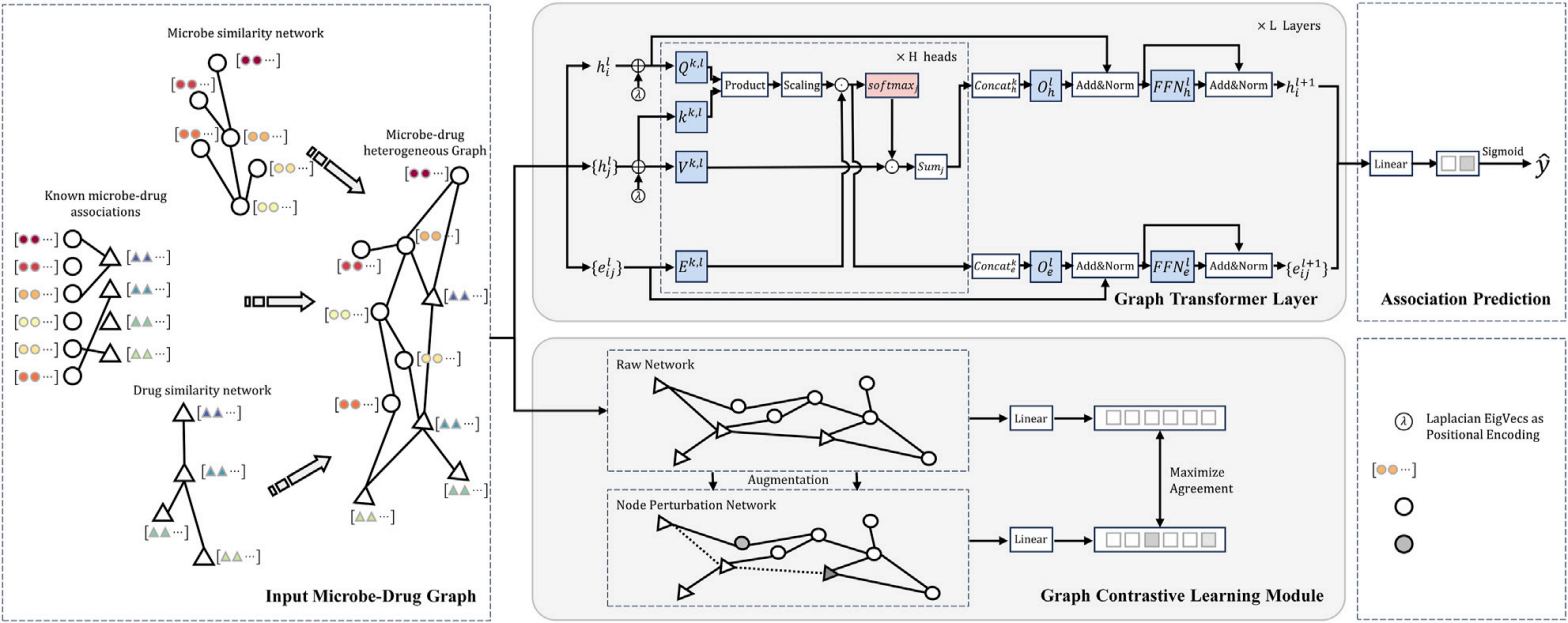
Structural diagram of the model proposed in this study for the microbe-drug association prediction task.

Figure 1 shows the detailed architecture of the Graph Contrastive Learning Model with Transformer proposed in this study for Microbe-Drug Associations Prediction (abbreviated as CLMT). The model aims to capture underlying structural relationships in microbe-drug graphs and enhance the robustness and discriminative power of the representation through contrastive learning. The CLMT model consists of four main modules: the Input Microbe-Drug Graph, the Graph Transformer Module, the Graph Contrastive Learning Module, and the Association Prediction Network.

First, the model constructs a heterogeneous graph structure composed of microbes and drugs as input. This graph is then processed by the Graph Transformer Network to capture potential association relationships between the nodes (Yun et al., 2019). Next, the Graph Transformer encoder further refines these association relationships within the microbe-drug graph structure. The model also incorporates contrastive learning (You et al., 2020), generating multiple augmented views of the graph as positive samples, while negative samples are derived from different graphs. By calculating the contrastive loss between the original graph and its augmented views, the model learns a more robust representation. Finally, the Association Prediction Network formalizes this task as a binary classification problem to compute the potential association information between microbes and drugs.

### 2.3 Input microbe-drug graph

The Microbe-Drug Graph Representation Layer is the base module of the CLMT model. The layer receives raw microbe and drug data, transforms them into heterogeneous graph structures and computes the similarity matrices of drugs and microbes, and finally generates microbe-drug embedding representations. These embeddings representations are used as input vectors for the subsequent graph Transformer learning module.

The initial inputs to this layer are raw microbial data and drug data. First, based on known microbe-drug associations, we construct a heterogeneous network structure by combining drug similarity and microbe similarity networks. We define the microbe-drug neighbor matrix $\in R^{n_{d}\times n_{m}}$ , where $n_{m}$ and $n_{d}$ denote the number of microbes and drugs, respectively. If the first i the drug $d_{j}$ is associated with the number of j microorganism $m_{j}$ there is an association between them, then the element at the corresponding position in the adjacency matrix $A_{ij}$ takes the value of 1, otherwise it takes the value of $A_{ij}=0$ .

To calculate the similarity of microbial nodes as well as drug nodes, we introduce exponential similarity. Exponential similarity is a method commonly used to calculate similarity between nodes (Goodall, 1966). Setting $A\left(d_{i}\right)$ and $A\left(m_{j}\right)$ denote the adjacency matrix respectively A the rows of i rows and j columns, then the drugs ${\;d}_{i}$ and ${\;d}_{j}$ The exponential similarity between is calculated as follows:

$$S_{d}^{\exp}\left(d_{i},d_{j}\right)=\exp\left(-\beta ∥A\left(d_{i}\right)-A\left(d_{j}\right){∥}^{2}\right)$$

$$\beta =\frac{\beta^{\prime }}{\left(\frac{1}{n_{d}}\sum_{i=1}^{n_{d}}\;∥A\left(d_{i}\right){∥}^{2}\right)}$$

Where $\beta^{\prime }$ is the tuning parameter and takes the value of 1. $\left\|*\right\|$ denotes the Frobenius norm. Similarly, the exponential similarity matrix of microorganisms ${\;S}_{m}^{\exp}$ is calculated similarly:

$$S_{m}^{\exp}\left(m_{i},m_{j}\right)=\exp\left(-\beta ∥A\left(m_{i}\right)-A\left(m_{j}\right){∥}^{2}\right)$$

$$\beta =\frac{\beta^{\prime }}{\left(\frac{1}{n_{m}}\sum_{i=1}^{n_{m}}\;∥A\left(m_{i}\right){∥}^{2}\right)}$$

Next, we use Jaccard Similarity (Bag S et al., 2019) to measure the similarity between nodes. Jaccard similarity measures similarity based on the ratio of intersection to concatenation. The Jaccard similarity between drug node pairs is defined as follows:

$$S_{d}^{Jac}\left(d_{i},d_{j}\right)=\frac{\left.\left|A\right.\left(d_{i}\right)\cap A\left(d_{j}\right)\right|}{\left.\left|A\right.\left(d_{i}\right)\cup A\left(d_{j}\right)\right|}$$

where $\left|*\right|$ denotes the number of elements in the set. Similarly, we have calculated the Jaccard similarity of microorganisms:

$$S_{m}^{Jac}\left(m_{i},m_{j}\right)=\frac{\left.\left|A\right.\left(m_{i}\right)\cap A\left(m_{j}\right)\right|}{\left.\left|A\right.\left(m_{i}\right)\cup A\left(m_{j}\right)\right|}$$

Further, we combine the index similarity of drugs $S_{d}^{\exp}$ and Jaccard Similarity $S_{d}^{Jac}$ , to get the integrated drug similarity matrix $S_{d}$ :

$$S_{d}=\frac{S_{d}^{\exp}+S_{d}^{Jac}}{2}$$

Similarly, the integrated microbial similarity matrix $S_{m}$ is calculated as follows:

$$S_{m}=\frac{S_{m}^{\exp}+S_{m}^{Jac}}{2}$$

Ultimately, we construct graph networks based on these integrated similarity and adjacency matrices:

$$N=\left[\begin{array}{cc} S_{d} & A\\ A^{T} & S_{m} \end{array}\right]$$

The graph network constructed in this way N that not only retains the similarity information of drugs and microorganisms, but also incorporates the interactions between them, providing a rich feature representation for subsequent graph neural network models.

### 2.4 Graph transformer module

In this study, the Graph Transformer module is the core component, which is designed to capture potential microbe-drug association features by learning the deep representation of nodes in the microbe-drug graph structure.

The graph attention mechanism is the key mechanism of the Graph Transformer module, which allows nodes to dynamically adjust their own representations in microbial-drug networks by taking into account the information of neighboring nodes (Wang X et al., 2019). Specifically, in the first l layer, the feature representation of each node is $H^{\left(l\right)}\in R^{\left(n_{d}+n_{m}\right)\times d^{\left(l\right)}}$ , where $n=n_{d}+n_{m}$ is the total number of nodes, i.e., the sum of the number of microbes and drugs, and $d^{\left(l\right)}$ is the number of nodes in the first l number of hidden units in the layer. And the node features can be obtained by linear transformation:

$$Z^{\left(l\right)}=H^{\left(l\right)}W^{\left(l\right)}$$

where $W^{\left(l\right)}\in R^{d^{\left(l\right)}\times d^{\left(l\right)}}$ is the learnable weight matrix. Multihead attention allows the model to learn multiple sets of different attention weights in parallel to capture the relationships of different feature subspaces in a heterogeneous network. For the first l layer of multi-head attention, node i and its neighbor nodes j The attention weights between the node and its neighbor nodes $\alpha_{ij}^{\left(l,k\right)}$ can be computed in the following way:

$$\alpha_{ij}^{\left(l,k\right)}=\frac{\exp\left(LeakyReLU\left(a^{{\left(l,k\right)}^{T}}\left[Z_{i}^{\left(l\right)}∥Z_{j}^{\left(l\right)}\right]\right)\right)}{\sum_{j^{\prime }\in N_{i}}\;\exp\left(LeakyReLU\left(a^{{\left(l,k\right)}^{T}}\left[Z_{i}^{\left(l\right)}∥Z_{j^{\prime }}^{\left(l\right)}\right]\right)\right)}$$

where $a^{\left(l,k\right)}\in R^{2d^{\left(l\right)}}$ is the learned attention parameter, and $LeakyReLU\left(*\right)$ is the activation function, and $N_{i}$ denotes the node i the set of neighbors of the node, and ∥ denotes the vector splicing operation. Further, the Graph Transformer module introduces a multi-head attention mechanism, where the outputs of multiple attention heads undergo a splicing operation to obtain the final node representation:

$$H_{i}^{\left(l+1\right)}=Concat\left(\left[H_{i}^{\left(l+1,1\right)},\ldots ,H_{i}^{\left(l+1,K\right)}\right]\right)$$

where K is the number of attention heads. In addition, in order to promote the stability of information transfer and model training, the output of each layer of Graph Transformer is subjected to residual concatenation and layer normalization, a process that can be formally described as:

$$H_{i}^{\left(l+1\right)}=LayerNorm\left(H_{i}^{\left(l\right)}+H_{i}^{\left(l+1\right)}\right)$$

where $\text{LayerNorm}\left(*\right)$ denotes the layer normalization operation.

After iterative updating by the multi-layer graph attention mechanism, the feature representation of each node in the final layer of the microbe-drug network $H^{\left(L\right)}$ contains rich feature information of drugs and microbes, which can effectively capture the potential interaction patterns and association laws between them.

### 2.5 Graph contrastive learning module

The Graph Contrastive Learning module is designed to enhance the model’s ability to extract informative and discriminative representations of nodes in the heterogeneous microbe-drug interaction network. By leveraging contrastive learning, our model learns to maximize the agreement between positive samples (different augmented views of the same node) while minimizing the similarity with negative samples (nodes from different distributions).

In order to enhance the model’s understanding of the structure of the microbe-drug graph and to improve the generalization ability of the overall model, we employed graph data augmentation techniques to generate multiple augmented views of the original graph, thereby enriching the data sample space for model training. Specifically, we used the node perturbation method to generate augmented graphs (Hiratani N et al., 2022). For each node in the microbe-drug graph structure, we randomly perturbed its feature vector to simulate the variation and uncertainty of node features. Let the node in the original graph i of the original graph be represented by the features of $h_{i}$ , the feature representation of the node after node perturbation is ${\tilde{h}}_{i}$ , the node perturbation process can be formalized as:

$${\tilde{h}}_{i}=h_{i}+\epsilon_{i},\epsilon ∼N\left(0,\sigma^{2}\right)$$

where $\epsilon_{i}$ is a random perturbation added to the node features, usually obeying some predefined distribution such as Gaussian or uniform. In this way, with the node perturbations, we can generate multiple augmented views with a slightly different structure from the original graph.

The purpose of the node perturbation operation is to introduce enough randomness to increase the diversity of the data and thus help the model learn a representation that is robust to noise and variation in the input data. In the graph contrast learning framework, these augmented views are used as the basis for the generation of positive sample pairs for optimizing the contrast learning process of the model. For the multiple augmented views generated, further inputs are provided to learn the deep feature representation of the nodes in Graph Transformer. For the output of Graph Transformer, the high-dimensional node representations are mapped to a low-dimensional space suitable for comparative learning through Feature Transformation. The goal of Feature Transformation is to reduce the dimensionality of the representations and to enhance their expressive power, typically using a fully connected layer, a process that can be formalized as:

$$z_{i}=FeatureTransformation\left(H_{i}^{\left(L\right)}\right)$$

where ${\;H}_{i}^{\left(L\right)}$ is the first L node representation of the layer, and $z_{i}$ is the node representation after projection.

After generating augmented views, we apply a contrastive loss function to maximize agreement between the original and augmented representations while ensuring separation from negative samples. Specifically, for each node in the microbe-drug graph structure $z_{i}$ , we define its contrast loss as:

$$L_{con}=-\log\frac{\exp\left(\sin\left(z_{i},z_{j}\right)/\tau \right)}{\sum_{k=1}^{2N}\;1_{\left[k\neq i\right]}\exp\left(\sin\left(z_{i},z_{k}\right)/\tau \right)}$$

$$\sin\left(z_{i},z_{j}\right)=\frac{z_{i}\cdot z_{j}}{∥z_{i}∥∥z_{j}∥}$$

where $\sin\left(z_{i},z_{j}\right)$ are the nodes $z_{i}$ and $z_{j}$ the similarity between the nodes, and τ is the temperature parameter, which controls the sharpness of the similarity distribution. $1_{\left[k\neq i\right]}$ denotes the indicator function that ensures the sum normalization of pairs of samples other than positive samples. One critical aspect of contrastive learning is the selection of negative samples, as poorly chosen negatives can lead to suboptimal representations. We employ semi-hard negative mining, where negative samples are selected based on their similarity scores. Nodes with extremely low similarity are ignored, as they contribute little useful information. Nodes with moderate similarity are prioritized, as they force the model to learn more discriminative features.

The loss function optimizes the embeddings such that positive pairs (nodes representing the same entity in different augmentations) are pulled closer together, while negative pairs (nodes from different distributions) are pushed apart.

The graph contrast learning module combines graph data enhancement and unsupervised contrast learning ideas to effectively optimize the representation learning process of microbial-drug graphs, and experimental results show that the module can improve the model’s prediction accuracy and generalization ability of microbial-drug associations.

### 2.6 Association prediction network

In the association prediction layer, we will utilize the microbial and drug graph structure representations obtained from the prelude steps for association prediction. Since the output of the model is still the node representations learned by Graph Transformer and Graph Comparison Learning Module, we first map these high-dimensional node representations to the final association prediction results. Specifically, we reduce the set of nodes by a linear transformation Z for dimensionality reduction, and the linear transformation can be expressed as:

H=ZW+b

where H is the node representation matrix after linear transformation. Then, the sigmoid activation function is utilized to map the linearly transformed node representations into the $\left[0,1\right]$ the probability space for predicting the association probability between microorganisms and drugs:

$$\hat{Y}=\sigma \left(H\right)$$

Where. $\hat{Y}\in R^{1}$ denotes the probability of potential association between microorganism and drug.

### 2.7 Loss function

**FIGURE 2:**
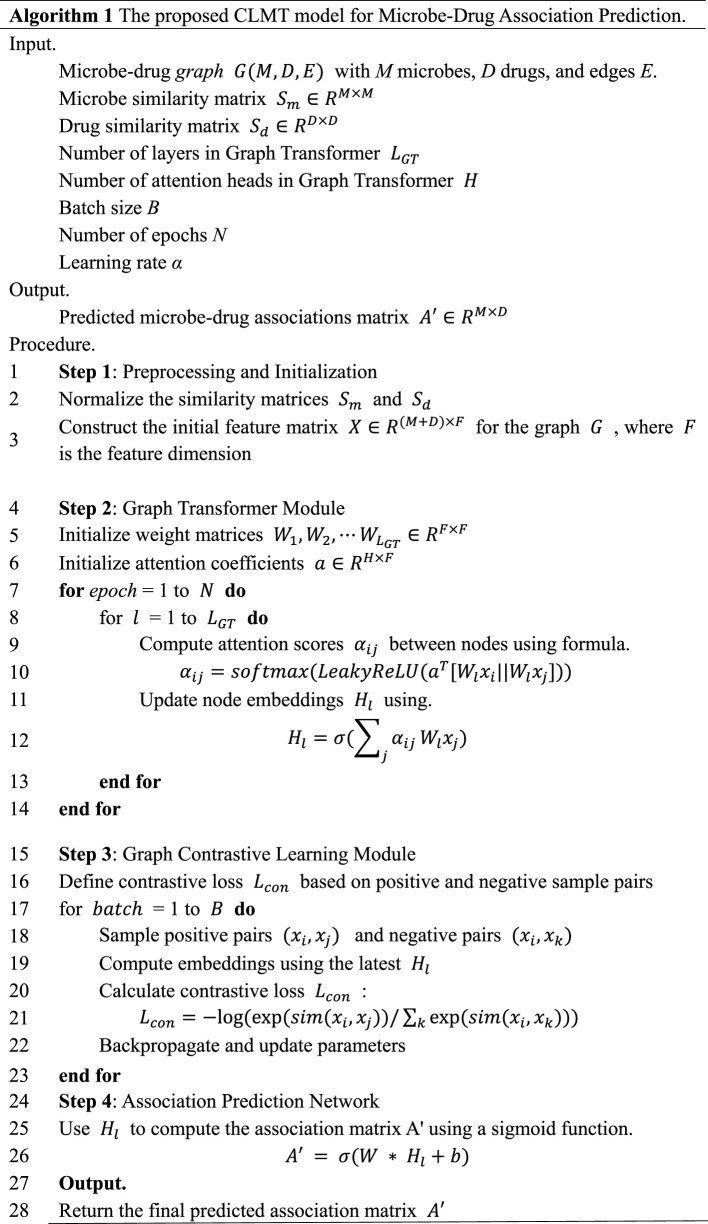
Pseudocode of the CLMT model proposed in this study.

At this point, we complete the inference process of the CLMT model, and the pseudo-code corresponding to this process is shown in Figure 2. In order to measure the difference between the predicted and true values of the model, we use the cross-entropy loss function to evaluate the effect of microbe-drug association prediction (Mao A et al., 2023). The cross-entropy loss function is a commonly used loss function in classification problems, and in microbe-drug association prediction, we modeled the problem as a binary classification task, i.e., predicting whether a certain pair of microbes and drugs are associated. The cross-entropy loss function is defined as follows:

$$L_{CE}=-\frac{1}{N}\sum_{i=1}^{N}\left[y_{i}\log\left({\hat{y}}_{i}\right)+\left(1-y_{i}\right)\log\left(1-{\hat{y}}_{i}\right)\right]$$

where N denotes the sample size; $y_{i}$ is the first i true label of the first sample, which indicates the presence of association, and 0 indicates the absence of association. ${\hat{y}}_{i}$ is the model’s predicted probability for the i sample, indicating the probability of an association between the microbe and the disease. The cross-entropy loss function improves the accuracy of the prediction by penalizing the wrong prediction of the model so that the model continuously adjusts the parameters during the training process.

To prevent model overfitting, we add a regularization term to the loss function. The regularization term improves the generalization ability of the model by adding a penalty to the model complexity in the loss function, encouraging the model to choose simpler parameter configurations (Kukačka J et al., 2017). In the CLMT model, we use L2 regularization, i.e., weight decay. It is defined as follows:

$$L_{\text{reg}\;}=\lambda \sum_{k}{∥W_{k}∥}_{2}^{2}$$

Where λ is the number, which controls the weight of the regularization term; $W_{k}$ denotes the model’s first k weight matrix; ${∥W_{k}∥}_{2}^{2}$ is the number of $W_{k}$ the L2 paradigm of the sum of squares of the weight matrices. The regularization term prevents the model from overfitting the training data by penalizing excessively large values of the weights, thus improving the model’s performance on the test data.

Ultimately, the combined loss function of the CLMT model consists of an unsupervised graph-contrast learning loss, a cross-entropy loss, and a regularization term of the following form:

$$L=L_{con}+L_{CE}+L_{\text{reg}\;}$$

This comprehensive loss function optimizes the node representation in the microbe-drug graph structure on the one hand, and takes into account the accuracy of the model prediction and the complexity of the model to ensure that the model not only can accurately fit the training data during the training process, but also has good generalization ability.

## 3 Experiments and results

This section provides a comprehensive description of the experimental setup, evaluation metrics, and baseline methods used to assess the performance of the CLMT model. We also present the results of the experiments along with a detailed analysis. The effectiveness and superiority of the CLMT model in predicting microbe-disease associations are demonstrated through comparisons with several baseline methods. Additionally, Figure 2 presents the corresponding pseudo-code of the CLMT model.

### 3.1 Experimental setup

In this study, we extracted drug features, microbial characteristics, and microbe-drug association matrices from the MDAD and aBiofilm databases. These feature matrices were subsequently used to construct heterogeneous networks that represent the interactions between drugs and microbes.

For the CLMT model, we set the number of epochs to 1,000 and the learning rate for the optimization algorithm to 0.001. The Graph Transformer model was configured with 3 layers, while the Multihead Self-Attention module contained 6 heads. Specifically, we tested different configurations of the Graph Transformer layer (ranging from 1 to 5 layers) and found that using 3 layers achieved the best balance between model complexity and performance. A smaller number of layers (e.g., 1 or 2) led to insufficient representation learning, while a larger snumber of layers (e.g., 4 or 5) caused overfitting and increased computational costs without significant performance improvements. For the multi-head attention mechanism, we experimented with different head numbers (ranging from 2 to 8). We found that 6 heads provided the most effective feature aggregation, allowing the model to capture diverse interaction patterns between microbes and drugs. Using fewer heads (e.g., 2 or 4) limited the model’s ability to focus on multiple aspects of the relationships, while using more heads (e.g., 8) led to increased computational overhead without notable gains in predictive accuracy. In the contrastive learning module, we set the temperature parameter τ in the contrastive loss function to 0.5, following extensive empirical analysis. The temperature parameter controls the sharpness of the similarity distribution, affecting how the model distinguishes positive and negative pairs. To determine the optimal value of, we tested values in the range [0.1,1.0] with a step size of 0.1. We observed that smaller values ( τ <0.3) led to over-concentration of representations, where the model assigned overly confident similarity scores, reducing the discriminative ability of learned embeddings. Larger values ( τ >0.7) resulted in overly smooth embeddings, making it harder for the model to effectively separate positive and negative pairs. Setting τ = 0.5 achieved the best balance between representation compactness and separability, ensuring that positive pairs remained close while maintaining sufficient distinction from negative pairs.

To improve model generalization, we incorporated a stochastic deactivation strategy in the association prediction module of the linear layer, with a dropout rate of 50%.

The model was trained using the Adam optimizer with a weight decay prevent overfitting. We applied an early stopping criterion with a patience of 20 epochs, monitoring the validation loss to avoid unnecessary training cycles. During both training and evaluation, we performed multiple rounds of cross-validation. Specifically, 5-fold cross-validation was applied, where the dataset was randomly split into five subsets. In each fold, one subset was used as the test set, and the remaining four were used for training. To ensure the reliability and robustness of the results, the entire experiment was repeated five times, and the average performance metrics were reported. All experiments were conducted on a NVIDIA 2080Ti GPU (11GB VRAM). The GPU acceleration significantly improved the efficiency of graph-based operations, particularly in the Graph Transformer module and contrastive learning calculations.

### 3.2 Evaluation indicators

In order to evaluate the methodology proposed in this paper, we employ a series of evaluation metrics to comprehensively measure the performance of the model, including AUC, AUPR and Accuracy. The following are the formal definitions and calculations of each evaluation metric:

AUC (Area Under the ROC Curve) represents the area under the receiver operating characteristic curve (ROC Curve), which is used to measure the classification performance of the model. The ROC Curve plots the True Positive Rate (TPR) and False Positive Rate (FPR) through different thresholds. TPR and FPR are defined as follows:

$$\text{TPR}\left(\text{Recall}\right)=\frac{TP}{TP+FN}$$

$$\text{FPR}=\frac{FP}{FP+TN}$$

where TP denotes true positives (True Positives) and FP denotes False Positives, and FN denotes False Negatives, and TN denotes True Negatives. AUC is a threshold-independent metric, meaning it evaluates model performance across all possible decision thresholds rather than a single threshold. It measures the model’s discrimination ability—the probability that a randomly chosen positive sample is ranked higher than a randomly chosen negative sample. In tasks like microbe-drug association prediction, where both false positives (misidentifying non-associations as associations) and false negatives (failing to identify true associations) are critical, AUC provides a balanced view of the model’s classification performance.

AUPR (Area Under the Precision-Recall Curve) denotes the area under the Precision-Recall Curve, which is used to measure the classification performance of the model on unbalanced datasets. The Precision-Recall Curve plots Precision and Recall through different thresholds. Precision and Recall are defined as follows:

$$Precision=\frac{TP}{TP+FP}$$

$$\text{Recall}=\frac{TP}{TP+FN}$$

where TP denotes true positives (True Positives) and FP denotes False Positives, and FN denotes False Negatives. Precision reflects the proportion of samples predicted to be positive by the model that are actually positive, while recall reflects the proportion of samples that are actually positive that are correctly predicted to be positive. AUPR has a value between 0 and 1, with larger values indicating better model performance. Since microbe-drug association datasets often contain significantly more negative samples than positive ones, AUC may overestimate model performance by giving equal weight to both classes. AUPR, on the other hand, focuses on the positive class and better reflects the model’s ability to identify meaningful associations.

In addition to AUC and AUPR, we also report Accuracy as a standard evaluation metric to measure the overall correctness of the model’s predictions. Accuracy is defined as:

$$Accuracy=\frac{TP+TN}{TP+TN+FP+FN}$$

Accuracy provides a simple and intuitive measure of the model’s classification ability. It is useful when the dataset is relatively balanced, as it evaluates both positive and negative class predictions equally.

### 3.3 Methods of comparison

To evaluate the performance of our proposed method, we compared it with five existing microbe-drug association prediction approaches. A brief overview of each method and their limitations is provided below:

HMDAKATZ (Zhu et al., 2019): This method predicts microbe-drug associations using the KATZ metric. However, it primarily relies on traditional graph metrics, which are unable to capture complex long-range dependencies. It also fails to account for important biological features of microbes and drugs, such as drug side effects, which limits its applicability to novel drugs or microbes with unknown associations.

GCNMDA (Long et al., 2020b): This method is based on Graph Convolutional Networks (GCNs) and conditional random fields to predict associations between microbes and drugs. While GCNs can capture local interactions, they struggle to model complex heterogeneous network structures and long-range dependencies, which affects their performance in handling noisy data and unknown associations.

GSAMDA (Tan et al., 2022): GSAMDA uses graph attention networks and sparse autoencoders to model both topological and attribute features within a microbe-drug heterogeneous network. However, its performance is limited by data sparsity, especially when there is insufficient labeled data, and it does not adequately model the intricate biological interactions between microbes and drugs.

LAGCN (Yu et al., 2021): LAGCN applies graph convolution to learn drug and disease embeddings, using an attentional mechanism to integrate embeddings from multiple layers for drug-disease association prediction. However, it is optimized for drug-disease predictions and does not specifically target microbe-drug associations, limiting its effectiveness for the task at hand.

NTSHMDA (Luo and Long, 2018): This method uses an improved randomized roaming algorithm to infer microbe-disease associations by integrating topological similarities within a microbe-drug network. However, it overlooks important biological features such as microbial genome information and drug side effects, which reduces its predictive power, especially for microbe-drug interactions.

These methods were evaluated on the MDAD, aBiofilm and Drug Virus datasets, using their default configurations and tuning their hyperparameters. All methods underwent 5-fold cross-validation, with known microbe-drug associations serving as positive samples and randomly generated negative samples for the training and test sets. To minimize sampling bias, each comparison was repeated five times, and the final AUC score was reported as the average of these iterations.

In contrast to these methods, our CLMT model introduces several innovations:

1. Graph Transformer Network: CLMT uses a Graph Transformer network to capture complex, long-range dependencies within the microbe-drug interaction network, surpassing the limitations of GCN-based approaches.
2. Contrastive Learning: By leveraging contrastive learning and generating multiple augmented views of the graph, CLMT significantly improves the model’s ability to learn discriminative and generalizable embeddings, even with sparse data.
3. Prediction of Novel Interactions: CLMT excels at predicting not only known associations but also novel microbe-drug interactions, making it more versatile and applicable in real-world scenarios where data may be limited or incomplete.

Our extensive experiments on the MDAD, aBiofilm and Drug Virus datasets demonstrate that CLMT outperforms these existing methods, offering superior predictive accuracy and uncovering novel microbe-drug associations with greater reliability.

### 3.4 Experimental results and analysis

Tables 2, 3 present the AUC, AUPR, and Accuracy scores of the CLMT model proposed in this paper, along with those of the compared methods on the MDAD and aBiofilm datasets. As shown in the tables, the CLMT method achieved the highest AUC (0.9735 and 0.9742), AUPR (0.9720 and 0.9714), and Accuracy (0.9045 and 0.9121) scores on both datasets, significantly outperforming the other five methods.

**TABLE 2:** 5-fold cv results on MDAD dataset.

Model	AUC	AUPR	Accuracy
HMDAKATZ	0.8712 ± 0.0010	0.8798 ± 0.0068	0.7691 ± 0.0167
GCNMDA	0.9365 ± 0.0001	0.9300 ± 0.0002	0.8617 ± 0.0011
GSAMDA	0.9460 ± 0.0197	0.9223 ± 0.0164	0.7979 ± 0.0279
LAGCN	0.8974 ± 0.0056	0.9062 ± 0.0050	0.8572 ± 0.0067
NTSHMDA	0.8512 ± 0.0043	0.8094 ± 0.0055	0.7820 ± 0.0137
CLMT	0.9735 ± 0.0014	0.9720 ± 0.0025	0.9045 ± 0.0031

**TABLE 3:** 5-fold cv results on aBiofilm dataset.

Model	AUC	AUPR	Accuracy
HMDAKATZ	0.8982 ± 0.0042	0.9018 ± 0.0037	0.7811 ± 0.0083
GCNMDA	0.9465 ± 0.0073	0.9376 ± 0.0026	0.8772 ± 0.0012
GSAMDA	0.8955 ± 0.0020	0.9073 ± 0.0033	0.8345 ± 0.0001
LAGCN	0.8991 ± 0.0047	0.9084 ± 0.0028	0.8710 ± 0.0011
NTSHMDA	0.8633 ± 0.0065	0.8204 ± 0.0045	0.8073 ± 0.0038
CLMT	0.9742 ± 0.0024	0.9714 ± 0.0011	0.9121 ± 0.0005

First, several comparative methods have demonstrated effectiveness in microbe-disease association tasks. For instance, the GSAMDA model, which utilizes graph attention networks and sparse autoencoders, achieved AUC scores of 0.9460 and 0.8955, and AUPR scores of 0.9223 and 0.9073 on the MDAD and aBiofilm datasets, respectively. These results indicate that GSAMDA effectively captures the topological and attribute features of nodes in the newly constructed microbial-drug heterogeneous network. Specifically, when dealing with graph data involving complex relationships, the graph attention network (GAT) can effectively focus on important node features through the attention mechanism, while the sparse autoencoder (SAE) can capture the data’s sparse structure. These characteristics enable GSAMDA to perform well in this task, demonstrating the feasibility of using graph neural networks and autoencoders for microbe-drug association prediction.

However, despite the satisfactory performance of many methods on this task, their AUC, AUPR, and Accuracy metrics still have room for improvement. Taking the GSAMDA model as an example, its AUC on the aBiofilm dataset is 0.8955, and its AUPR is 0.9073, which represents a gap of 5.05% and 1.5%, respectively, compared to its performance on the MDAD dataset. This gap suggests that GSAMDA has limitations, particularly when handling different datasets, indicating its potential shortcomings in capturing features and modeling relationships. Therefore, the microbe-drug association prediction task requires further exploration, and more powerful and robust methods are necessary to enhance prediction performance.

In comparison, the CLMT method proposed in this paper significantly outperforms all other methods on both datasets. The three evaluation metrics on the MDAD dataset are 0.9735, 0.9720, and 0.9045, respectively, while on the aBiofilm dataset, the corresponding metrics are 0.9742, 0.9714, and 0.9121. We attribute this superior performance to the unique model structure and design principles of CLMT.

To further verify whether the experimental results of CLMT are statistically significant, we conducted a statistical analysis on the AUC scores from 5-fold cross-validation and compared them with a baseline method. Since some existing methods do not have publicly available implementations, we reproduced GSAMDA, one of the best-performing models on the MDAD dataset, as a comparison model and computed the p-value to assess whether CLMT provides a statistically significant improvement. On the MDAD test set, the AUC scores from 5-fold cross-validation for GSAMDA were [0.9497, 0.9277, 0.9389, 0.9539, 0.9581], while our proposed CLMT achieved [0.9735, 0.9730, 0.9737, 0.9748, 0.9729] under the same conditions. To quantify whether the performance gain of CLMT over GSAMDA is statistically significant, we applied a paired t-test, obtaining a p-value of 0.0010 (p < 0.05). This result confirms that the improvement of CLMT over GSAMDA is not due to random variations but represents a statistically significant performance enhancement driven by the methodological improvements introduced in CLMT.

CLMT employs data enhancement techniques such as node perturbation, which enriches the training data by generating a multi-view graph structure. This technique helps the model better learn the diversity of nodes and edges within the graph, thereby improving its generalization ability. More importantly, in the graph contrastive learning module, CLMT utilizes a projection head to map the node representations output by the graph encoder to a space suitable for contrastive learning. By calculating the contrastive loss, this mechanism maximizes the consistency between different views of the same graph structure and minimizes the similarity between different graph structures. This contrastive learning mechanism effectively enhances the model’s ability to capture graph structure features, enabling it to make more accurate association predictions when faced with different graph structures.

Additionally, CLMT incorporates a Transformer model based on a multi-head self-attention mechanism within the graph encoder. This approach enhances the model’s representational capacity by capturing various relationships and feature interactions between nodes through multiple attention heads. The multi-head self-attention mechanism not only focuses on globally important features but also mitigates the overfitting problem that can arise from relying on a single attention head.

Table 4 presents the AUC, AUPR, and Accuracy scores of the CLMT model proposed in this paper, along with those of the compared methods on the Drug Virus dataset. As shown in the table, the CLMT method achieved the highest AUC (0.9727), AUPR (0.9699), and Accuracy (0.9235), significantly outperforming the other five methods.

**TABLE 4:** 5-fold cv results on Drug Virus dataset.

Model	AUC	AUPR	Accuracy
HMDAKATZ	0.8523 ± 0.0074	0.8617 ± 0.0045	0.7245 ± 0.0074
GCNMDA	0.9214 ± 0.0052	0.8965 ± 0.0037	0.8674 ± 0.0069
GSAMDA	0.8754 ± 0.0024	0.8868 ± 0.0064	0.8745 ± 0.0068
LAGCN	0.9214 ± 0.0036	0.9247 ± 0.0029	0.8958 ± 0.0036
NTSHMDA	0.8354 ± 0.0085	0.8004 ± 0.0074	0.7954 ± 0.0023
CLMT	0.9727 ± 0.0012	0.9699 ± 0.0014	0.9235 ± 0.0007

Despite the reasonable performance of many existing methods, their AUC, AUPR, and Accuracy metrics still have room for improvement. For example, the GSAMDA model, which utilizes graph attention networks and sparse autoencoders, achieved an AUC of 0.8754 and an AUPR of 0.8868 on the Drug Virus dataset. While GSAMDA successfully captures node attributes and sparse structures, its performance lags behind that of CLMT, highlighting potential limitations in generalizing to diverse datasets. Similarly, the HMDAKATZ model, based on heterogeneous graph diffusion, showed the lowest performance, with an AUC of 0.8523 and an Accuracy of 0.7245, indicating its struggles in capturing complex relationships in Virus-drug interactions.

In comparison, the CLMT method proposed in this paper significantly outperforms all other methods across all evaluation metrics. The three evaluation metrics on the Drug Virus dataset are 0.9727, 0.9699, and 0.9235, respectively. We attribute this superior performance to the unique model structure and design principles of CLMT.

Overall, the results on the Drug Virus dataset further validate the effectiveness of CLMT in microbial-drug association prediction. By leveraging contrastive learning, self-attention mechanisms, and data augmentation techniques, CLMT demonstrates superior adaptability and generalization capabilities, setting a new benchmark for future research in this domain.

### 3.5 Ablation experiment

**FIGURE 3:**
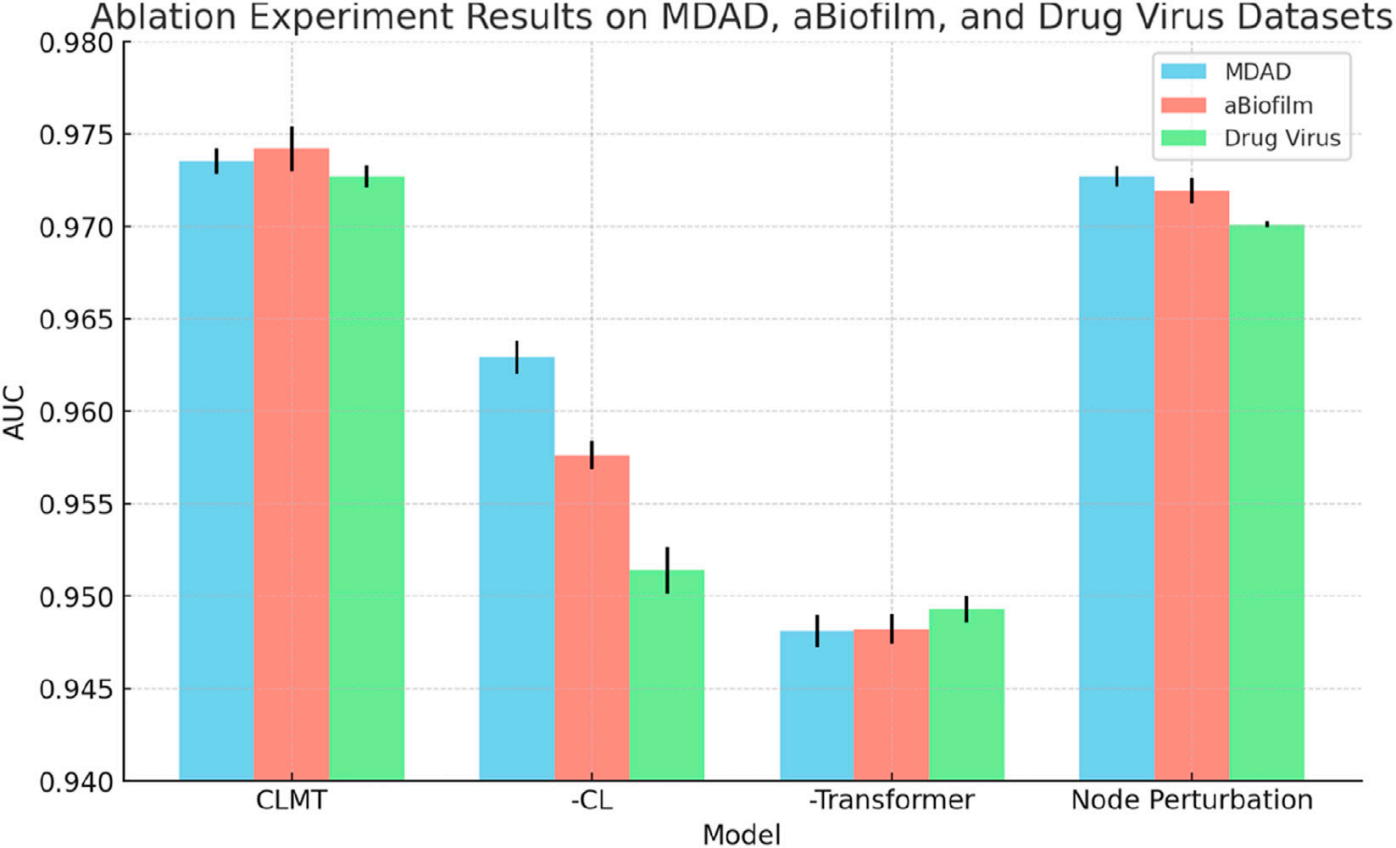
Performance comparison of CLMT, CLMT-CL, CLMT-Transformer, CLMT-node perturbation on MDAD, aBiofilm, and DrugVirus datasets.

To further validate the effectiveness of the individual modules in our proposed CLMT method, we conducted ablation experiments on the MDAD, aBiofilm, and DrugVirus datasets. The results are shown in Table 5 and Figure 3.

**TABLE 5:** Results of ablation experiments on MDAD, aBiofilm, and DrugVirus datasets.

Model	AUC		
	MDAD	aBiofilm	Drug virus
CLMT	0.9735 ± 0.0014	0.9742 ± 0.0024	0.9727 ± 0.0012
-CL	0.9629 ± 0.0018	0.9576 ± 0.0015	0.9514 ± 0.0025
-Transformer	0.9481 ± 0.0017	0.9482 ± 0.0016	0.9493 ± 0.0014
-node perturbation	0.9727 ± 0.0011	0.9719 ± 0.0014	0.9701 ± 0.0003

**FIGURE 4:**
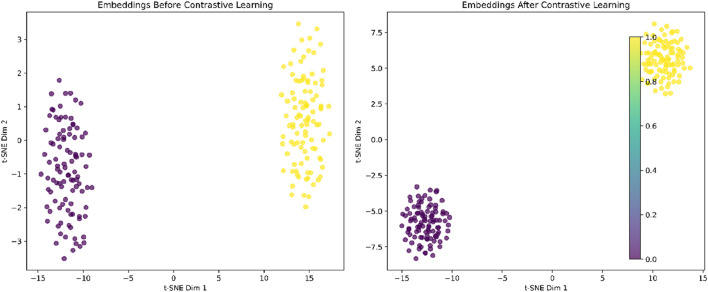
Effect of contrastive learning on embedding distributions.

When the Graph Contrastive Learning Module was removed, the model exhibited consistent performance degradation across all datasets. Specifically, the AUC decreased from 0.9735 to 0.9629 on MDAD, 0.9742 to 0.9576 on aBiofilm, and 0.9727 to 0.9514 on DrugVirus. These results highlight the critical role of contrastive learning in enhancing the model’s discriminative ability by maximizing consistency between augmented graph views. The significant performance drop (average 1.9% across datasets) underscores its contribution to generalization. Additionally, to qualitatively analyze the effectiveness of the contrastive learning module, we present the embedding distribution of the MDAD dataset’s test data. Specifically, we obtained the high-dimensional embeddings of the test data both “before” and “after” contrastive learning, performed clustering, and visualized the results using t-SNE, as shown in Figure 4. The left image shows the embeddings before contrastive learning, where clusters are present but may overlap due to large variance. The right image shows the embeddings after contrastive learning, where the clusters are more compact and distinctly separated, indicating improved feature discrimination.

Removing the Graph Transformer Module led to the most pronounced performance decline, with AUC values dropping to 0.9481 (MDAD), 0.9482 (aBiofilm), and 0.9493 (DrugVirus). This demonstrates the Transformer’s irreplaceable capability in modeling complex global dependencies and feature interactions within the graph structure. The multi-head self-attention mechanism effectively captures long-range relationships, which is particularly crucial for sparse biological networks like DrugVirus.

Replacing node perturbation with random edge deletion caused minor but consistent performance degradation across all datasets: AUC decreased to 0.9727 (MDAD), 0.9719 (aBiofilm), and 0.9701 (DrugVirus). While edge deletion remains a viable augmentation strategy, node perturbation’s superior performance (average 0.3% improvement) suggests its advantage in preserving critical structural information during view generation. This effect is especially notable on DrugVirus, where biological interaction sparsity demands more nuanced augmentation.

The ablation experiments confirm that each module uniquely enhances CLMT’s performance:Contrastive learning mitigates overfitting through view invariance. Graph Transformer enables global relational reasoning. Node perturbation optimizes augmentation for biological graph characteristics. Their combined effect achieves state-of-the-art AUC values (>0.97 on all datasets), validating CLMT’s robustness in diverse microbe-drug-virus association prediction scenarios.

### 3.6 Case study

In this case study, we aimed to validate the practical effectiveness of the CLMT model in identifying new microbe-drug associations by selecting three commonly used drugs-Cloxacillin, Carvacrol, and Ciprofloxacin-and the microorganism *Mycobacterium tuberculosis* from the MDAD dataset. For each drug, we cross-checked the top 20 predicted microorganisms by searching for their synonyms in the MeSH and DrugBank databases. Additionally, we verified whether the predicted microbe-drug associations had been reported in the scientific literature through PubMed searches.

Cloxacillin, a semi-synthetic penicillin antibiotic, is widely used to treat infections caused by beta-hemolytic streptococci, pneumococci, and staphylococci (Grillo et al., 2023). It is particularly effective against penicillinase-producing strains of *Staphylococcus aureus* and *Staphylococcus* epidermidis, which are resistant to other antibiotics (Aldman et al., 2022). Research has demonstrated that cloxacillin inhibits up to 50% of the activity of *S. aureus*, S. haematobium, and *Salmonella typhi* (Orogade and Akuse, 2004). In our study, 15 of the top 20 microorganisms predicted to be associated with cloxacillin (75%) were confirmed in the literature, as shown in Table 6.

**TABLE 6:** The top 20 Cloxacillin-related microbes predicted by CLMT and the related publications.

Rank	Microbe	Evidence
1	*Enterobacter* aerogenes	PMID22001269
2	*Clostridium* pasteurianum	Unconfirmeda
3	*Streptomyces* sp. nov.	PMID6970744
4	*Staphylococcus aureus*	PMID15490798
5	Burkholderia cepacia	Unconfirmeda
6	*Klebsiella pneumoniae*	PMID20597925
7	Thermus thermophilus	Unconfirmeda
8	*Bacillus subtilis*	PMID25945113
9	*Salmonella typhi*	PMID15490798
10	*Helicobacter pylori*	PMID10748053
11	Schistosoma	PMID15490798
12	*Candida* albicans	PMID2713774
13	*Micrococcus* luteus	PMID7771695
14	*Bacillus* cereusereus	PMID24876650
15	Francisella novicida	Unconfirmeda
16	Pantoea agglomerans	PMID33666040
17	*Candida* dubliniensis	PMID316353125
18	*Candida* spp.	PMID21496537
19	Baker’s yeast	PMID25945113
20	*Klebsiella* planticola	Unconfirmeda

Carvacrol, a naturally occurring phenolic monoterpene found in aromatic plants, has demonstrated a wide range of bioactivities in both *in vivo* and *in vitro* studies. These include antioxidant (Churklam et al., 2020), diabetes prevention (Arkali et al., 2021), hepatoprotective (Elbe et al., 2020), reproductive (Saghrouchni et al., 2023), antimicrobial, and immunomodulatory properties (Chraibi et al., 2020). Additionally, carvacrol is used as a food preservative due to its flavoring properties (Patel, 2015). Previous research has highlighted its association with various microorganisms. For instance (Abdelhamid and Yousef, 2021), described how carvacrol counteracts desiccation-resistant *Salmonella* nacionalis, suggesting its potential as an additive against desiccation-adapted *Enterococcus faecalis* in low-moisture foods (Javed et al., 2021). demonstrated that carvacrol and its metabolites have beneficial effects on immune dysfunction and infection related to COVID-19. Moreover (Wang Y. et al., 2020), found that carvacrol reduced biofilm formation and extracellular polysaccharide secretion by *Pseudomonas* fluorescens and *S. aureus*, without affecting cell viability. Of the top 20 microorganisms predicted to be associated with carvacrol, 17 were confirmed by the literature, as shown in Table 7.

**TABLE 7:** The top 20 Carvacrol-related microbes predicted by CLMT and the related publications.

Rank	Microbe	Evidence
1	*Streptococcus* mutans	PMID: 28233286
2	Enteric bacteria and other eubacteria	PMID: 16355827
3	*Streptomyces* sp. nov.	Unconfirmeda
4	*Vibrio* campbellii	Unconfirmeda
5	*Micrococcus* luteus	PMID: 33240953
6	*Salmonella enterica*	PMID: 20132667
7	*Staphylococcus aureus*	PMID: 34730626
8	Stenotrophomonas maltophilia	PMID: 14659660
9	*Enterococcus faecalis*	PMID: 29877104
10	*Bacillus anthracis*	Unconfirmeda
11	Kocuria rhizophila	PMID: 37481932
12	*Mycobacterium tuberculosis*	PMID: 31552700
13	*Klebsiella pneumoniae*	PMID: 34729712
14	Edwardsiella tarda	PMID: 37476823
15	*Pseudomonas aeruginosa*	PMID: 35776742
16	*Klebsiella* planticola	PMID: 23030501
17	*Staphylococcus* epidermidis	PMID: 37508194
18	Burkholderia cenocepacia	PMID: 26946055
19	*Salmonella Typhi*	PMID: 16355827
20	*Acinetobacter* baumannii	PMID: 25177730

Ciprofloxacin, a fluoroquinolone antibiotic, is widely used for treating a variety of infections, including pneumonia, typhoid fever, and skin and soft tissue infections (McCurdy et al., 2017). Numerous studies have confirmed its effectiveness against various human microorganisms. For example (Rehaman et al., 2019), demonstrated ciprofloxacin’s efficacy against *Pseudomonas aeruginosa*, an opportunistic pathogen (Liu et al., 2021). reported reduced lung inflammation in pneumonia patients treated with ciprofloxacin, while (Trinh et al., 2017) found that combining ciprofloxacin with ceftriaxone provided the most effective treatment for foodborne *Vibrio* traumaticus. In our study, all 20 of the top microorganisms predicted to be associated with ciprofloxacin were validated by the literature, as shown in Table 8.

**TABLE 8:** The top 20 Ciprofloxacin-related microbes predicted by CLMT and the related publications.

Rank	Microbe	Evidence
1	*Bacillus subtilis*	PMID: 33218776
2	*Mycobacterium tuberculosis*	PMID: 22421328
3	*Listeria* monocytogenes	PMID: 34068252
4	*Enterobacter cloacae*	PMID: 11909836
5	*Proteus* vulgaris	PMID: 34638966
6	Enteric bacteria and other eubacteri	PMID: 27436461
7	*Salmonella Typhi*	PMID: 31877141
8	Actinobacillus actinomycetemcomitans	PMID: 12019120
9	*Pseudomonas aeruginosa*	PMID: 30605076
10	*Micrococcus* luteus	PMID: 3010848
11	*Haemophilus* influenzae	PMID: 8453168
12	*Streptococcus* epidermidis	PMID: 27579011
13	*Staphylococcus aureus*	PMID: 35301951
14	*Klebsiella* planticola	PMID: 25465871
15	Providencia stuartii	PMID: 15528892
16	Stenotrophomonas maltophilia	PMID: 14982788
17	*Bacillus anthracis*	PMID: 22064542
18	*Escherichia coli*	PMID: 35091053
19	Porphyromonas gingivalis	PMID: 15231772
20	*Helicobacter pylori*	PMID: 25721770

In addition, *M. tuberculosis* was selected for our case study. This Gram-positive, aerobic bacterium is the causative agent of tuberculosis, one of the deadliest diseases worldwide. According to the 2019 Global Tuberculosis Report (WHO Global, 2019), tuberculosis resulted in 1.5 million deaths in 2018. As shown in Table 9, 17 of the top 20 predicted drugs for *M. tuberculosis* have been supported by prior studies. This underscores the CLMT model’s strong predictive ability in case studies involving drugs and microorganisms.

**TABLE 9:** The top 20 *Mycobacterium* tuberculosis-associated drugs predicted by CLMT and the related publications.

Rank	Drug	Evidence
1	Calanolide A	PMID: 14980631
2	Hydrogen peroxide	PMID: 30551469
3	Ciprofloxacin	PMID: 16154314
4	beta-Pinene	PMID: 19753839
5	Pyrazinamide	PMID: 26521205
6	Vitamin C	PMID: 23695675
7	Gentamicin	PMID: 22143521
8	Rilpivirine	Unconfirmeda
9	Ceforanide	PMID: 7624446
10	Zidovudine	PMID: 16154314
11	Polysorbate 80	Unconfirmeda
12	Amikacin	PMID: 29311078
13	Zinc oxide	PMID: 33845951
14	Vanillylacetone	Unconfirmeda
15	Vitamin E	PMID: 26491981
16	Darunavir	PMID: 28193650
17	Saquinavir	PMID: 33841429
18	Lopinavir	PMID: 21442799
19	Tobramycin	PMID: 19723387
20	Minocycline	PMID: 30597040

## 4 Discussion and conclusion

The microbe-drug association prediction task seeks to identify potential associations between microbes and drugs, which can support drug development and disease treatment. In this study, we propose the CLMT model for this task. The CLMT model improves learning capabilities by integrating a Graph Transformer network with contrastive learning techniques. Specifically, we utilize a multilayer Graph Convolutional Network (GCN) to capture the complex relationships between microbes and drugs. The contrastive learning module further enhances the model’s discriminative ability, thereby improving prediction accuracy.

By effectively modeling complex interactions and overcoming data sparsity, CLMT can serve as a valuable tool in early-stage drug screening, ultimately reducing experimental costs and speeding up the development pipeline. Its robust performance on public datasets suggests that CLMT has the potential to be integrated into clinical decision-making frameworks, offering insights that could lead to more personalized and effective treatment strategies. The findings of this study have notable biological implications. By elucidating previously unknown associations between microbes and drugs, CLMT can contribute to a deeper understanding of the molecular mechanisms underlying drug efficacy and resistance. These insights are particularly relevant in the context of rising antimicrobial resistance and the need for precision medicine. Furthermore, the ability of CLMT to highlight subtle, yet biologically meaningful patterns in microbe-drug interactions may inform future research on microbial metabolism, host-microbe interactions, and the role of the microbiome in disease progression. In this way, the model not only advances computational methodology but also holds promise for driving novel biological discoveries.

While our experimental results on two publicly available datasets demonstrate the effectiveness of CLMT, it is important to acknowledge several limitations and failure cases. In certain instances, the model’s performance was less robust. For example, in cases where the microbe-drug association data is extremely sparse, CLMT sometimes struggled to capture weaker or less obvious associations. This may be due to insufficient signal in the available data or limitations in the current data augmentation strategy. When the relationships between certain microbes and drugs are subtle or not well-characterized by the provided features, the model occasionally misclassified these associations. This suggests that additional biological information (e.g., gene expression profiles or metabolic pathways) might be needed to fully capture the underlying mechanisms. Although CLMT performs well on the MDAD and aBiofilm datasets, its scalability and effectiveness on larger or more heterogeneous datasets remain to be thoroughly evaluated. Future work is needed to optimize the model structure for such scenarios.

## Data Availability

The original contributions presented in the study are publicly available. This data can be found here: GitHub repository, https://github.com/qimou-515/CLMT.
